# Non-Rodent Genetic Animal Models for Studying Tauopathy: Review of *Drosophila*, Zebrafish, and *C. elegans* Models

**DOI:** 10.3390/ijms22168465

**Published:** 2021-08-06

**Authors:** Hoi-Khoanh Giong, Manivannan Subramanian, Kweon Yu, Jeong-Soo Lee

**Affiliations:** 1 Disease Target Structure Research Center, KRIBB, 125 Gwahak-ro, Yuseong-gu, Daejeon 34141, Korea; 097068@kist.re.kr (H.-K.G.); manivannan.s80@gmail.com (M.S.); 2 KRIBB School, University of Science and Technology, 125 Gwahak-ro, Yuseong-gu, Daejeon 34141, Korea; 3 Dementia DTC R&D Convergence Program, KIST, Hwarang-ro 14 gil 5, Seongbuk-gu, Seoul 02792, Korea

**Keywords:** tauopathy, pathophysiology, *Drosophila*, zebrafish, *C. elegans*, advantages, limitations

## Abstract

Tauopathy refers to a group of progressive neurodegenerative diseases, including frontotemporal lobar degeneration and Alzheimer’s disease, which correlate with the malfunction of microtubule-associated protein Tau (MAPT) due to abnormal hyperphosphorylation, leading to the formation of intracellular aggregates in the brain. Despite extensive efforts to understand tauopathy and develop an efficient therapy, our knowledge is still far from complete. To find a solution for this group of devastating diseases, several animal models that mimic diverse disease phenotypes of tauopathy have been developed. Rodents are the dominating tauopathy models because of their similarity to humans and established disease lines, as well as experimental approaches. However, powerful genetic animal models using *Drosophila*, zebrafish, and *C. elegans* have also been developed for modeling tauopathy and have contributed to understanding the pathophysiology of tauopathy. The success of these models stems from the short lifespans, versatile genetic tools, real-time in-vivo imaging, low maintenance costs, and the capability for high-throughput screening. In this review, we summarize the main findings on mechanisms of tauopathy and discuss the current tauopathy models of these non-rodent genetic animals, highlighting their key advantages and limitations in tauopathy research.

## 1. Introduction

Tauopathy refers to a group of neurodegenerative disorders that share a common feature of neurofibrillary tangles (NFT) originating from the aggregation of abnormal hyperphosphorylated and paired helical filament (PHF) Tau proteins [1]. The term tauopathy was first coined in 1997 to describe a familial dementia characterized by abundant and widespread intracellular NFT with the absence of amyloid β (Aβ) plaque deposits [2]. Since then, other neurodegenerative diseases have also been classified as tauopathy, including Alzheimer’s Disease (AD), frontotemporal dementia with Parkinsonism linked to chromosome 17 (FTDP-17), cortical basal degeneration, amyotrophic lateral sclerosis, dementia pugilistica, tangle-only dementia, progressive supranuclear palsy, and Pick’s disease [1,3].

Although the Tau protein was first identified almost a half century ago [4], scientists did not pay attention to this protein until the discovery of Tau as a major component of the abnormal aggregated tangles in AD patient brains a decade later [5,6,7,8,9]. Tau is one of the first characterized and well-known factors to maintain the stability of microtubules (MTs) in the central nervous system (CNS) [10,11]. Tau protein is subject to diverse post-translational modifications (PTMs), including glycosylation, glycation, nitration, polyamination, phosphorylation, ubiquitination, oxidation, cleavage or truncation, small ubiquitin-like modifier (SUMO) modification, and aggregation [12].

Thus far, 19 Tau-targeting therapeutic compounds have been proposed and tested based on diverse mechanisms by which Tau protein aggregation causes neurodegeneration. Unfortunately, all compounds have failed to pass phase 3 of clinical trials [13]. Therefore, tauopathies and AD, in particular, remain incurable diseases, and with the number of tauopathy-related dementias increasing every year, it is urgent to discover an effective therapeutic for tauopathy. In this review, we first summarize underlying mechanisms of tauopathy and then discuss research on tauopathy using non-rodent genetic animal models. We focus on *Drosophila*, zebrafish, and *C. elegans*, highlighting their advantages and limitations as tauopathy models in order to unveil mechanistic aspects of tauopathy and to develop novel therapeutic strategies. The most popular tauopathy model, the rodent model, has been extensively reviewed elsewhere [14] and is not covered here.

## 2. Underlying Mechanisms of Tauopathy

### 2.1. Normal Function of Tau

**Figure 1:**
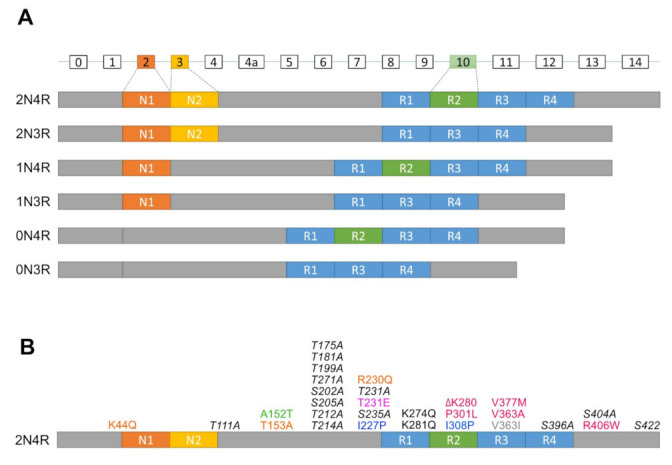
Diagrams of isoforms and mutations of the *MAPT* gene. (**A**) The *MAPT* gene contains 16 exons (rectangles with exon numbers on top), where exons with white rectangles are constitutive whereas exon 2 (dark orange box), exon 3 (orange box) and exon 10 (green box) are subject to alternative splicing. 0N, 1N, or 2N isoform depends on the presence of exon 2 and 3, while 3R or 4R isoform is determined by the presence of exon 10. (**B**) Mutations of the *MAPT* gene used to generate tauopathy models in *Drosophila*, zebrafish and *C. elegans*. dark orange, Calpain-resistant mutations; red, reduced microtubule-binding and increased aggregation mutations; green, a reduced microtubule-binding and increased oligomerization, but not fibrillization mutation; pink, an increased phosphorylation mutation; blue, anti-aggregation mutations; grey, promote microtubule assembly and increased oligomerization, but not fibrillization mutation; black, increased disease-associated lysine acetylation mutations; italic black, phosphorylation-incompetent mutations.

In humans, the Tau protein consists of six isoforms translated from alternatively spliced mRNAs of a single microtubule-associated protein Tau gene (*MAPT*) containing 16 exons (Figure 1A). These isoforms are classified by the combination of numbers of N (N-terminal projection) and R (microtubule-binding repeats) domains (i.e., 2N4R, 1N4R, 0N4R, 2N3R, 1N3R, and 0N3R). In particular, the number of N is determined by alternative splicing of exon 2 and 3 at the N terminal, whereas the number of R is defined by whether they contain repeat 2 encoded by exon 10 in mature mRNA [15,16]. In humans, mRNAs encoding 3R isoforms are found in both fetal and adult brains, whereas only mRNAs of 4R isoforms are found exclusively in the adult brain [15]. Moreover, the level of 3R and 4R isoforms in the adult brain is about equal to that which any disturbances are associated with neurodegeneration [17].

In general, Tau protein is ubiquitously expressed from the cell body to neurites in immature neurons but mainly found in the axon, interacting with MTs in mature neurons, where Tau regulates their stability [17]. However, Tau is also present in other cellular compartments, such as in both pre- and post-synaptic compartments [18,19]; at the post-synapse, Tau plays a role in establishing the proper localization of Fyn and is involved in regulating N-methyl-D-aspartate receptors (NMDARs) and post-synaptic density protein 95 (PSD95) complex [20,21]. In addition, Tau is found in the nucleus, where it binds to the heterochromatin and protects the integrity of DNA and RNA [22,23], and is localized in the neural plasma membrane in a MT-independent manner [24,25].

### 2.2. Phosphorylation of Tau

In AD and other tauopathies, phosphorylation of Tau is one of the main events among several PTMs that the Tau protein undergoes [26]. A 2N4R Tau usually carries approximately two phosphates per molecule [27]. Tau phosphorylation at Thr205, Thr231, and Ser396 is highly correlative with adult hippocampal neurogenesis as a compensatory mechanism to neuronal loss in AD [28]. In fact, similar Tau phosphorylation could reduce the endoplasmic reticulum (ER) stress and kinase-induced apoptosis [29,30]. However, in pathological conditions, the phosphorylation of Tau is approximately eight phosphates per molecule, an increase of three to four times more compared to normal individuals [6,26,31]. When hyperphosphorylated, Tau partially loses its binding affinity to MTs affecting their dynamics [32]. Hyperphosphorylated Tau also causes defects in axonal neurotransmitter transport, release, and uptake, thereby resulting in axon degeneration and finally neurodegeneration [33]. Therefore, Tau hyperphosphorylation is considered as an early-onset pathological process [34]. Known protein kinases and phosphatases that determine the degree of phosphorylation of Tau include glycogen synthase kinase 3β (GSK3β), mitogen-activated protein kinase (MAPK), cyclin AMP (cAMP)-dependent protein kinase A (PKA), calmodulin kinase II (CaMK II), protein kinase (PKC), microtubule affinity regulating kinase (MARK), and protein phosphatase family, especially type 2A (PP2A) [26,35].

In the longest isoform of Tau, 2N4R, there are approximately 85 potential phosphorylation sites (80 Serine or Threonine, and 5 Tyrosine residues). Among them, there are 17 Thr-Pro or Ser-Pro motifs that are abnormally hyperphosphorylated in AD and other Tauopathies [36], and the phosphorylation of Tau at Tyr394 and Tyr18 is detected in paired helix filaments (PHFs) in the brains of AD patients [37]. A study using the mouse tauopathy model also suggests that the hyperphosphorylation of Tau at tyrosine residues correlates with Tau aggregation [38]. Although all members of the protein phosphatase family are involved in dephosphorylation of Tau, PP2A is the major phosphatase responsible for almost 70% of the Tau phosphatase activity in human brains, and a loss of function of this protein phosphatase may be a cause of disease onset [36]. Consistently, PP2A activity is decreased by 20% to 40% in AD brains [39,40,41].

### 2.3. Aggregation of Tau

Both in vitro and in vivo studies have shown that Tau aggregation occurs in a “nucleation-elongation” mechanism [42]. The initiation of Tau aggregation can be affected by motif sequences, cofactors, phosphorylation status, and cleavage. In Tau protein, there are two small fractions at the beginning of R2 and R3 domains, with VQIINK and VQIVYK motifs, respectively. These hexapeptides are essential for forming β-sheet structures that tightly interlink the β-sheets together in a steric “zippers” manner, thereby leading to Tau aggregation [42,43]. Accordingly, the disruption or reinforcement of these motifs abolish or enhance the tendency of β-sheet structure and Tau aggregation, respectively [44]. Tau aggregation is also modulated by Tau cofactors. Negative-charged polyanions, such as glycosaminoglycans, and their sulfated forms (e.g., heparin) or RNA (e.g., polyU RNA) can promote Tau aggregation in vitro [45,46,47,48]. Another cofactor, FKBP52 (the 52 kD FK506-binding protein), also induces Tau aggregation in vitro and accelerates memory impairments in vivo [49,50].

In addition, among many post-translational modifications affecting Tau aggregation, Tau hyperphosphorylation was shown to precede aggregation significantly in patient samples and transgenic mice [51]. Such abnormal hyperphosphorylated Tau from AD brains can self-assemble into PHFs form in vitro [52]. However, it has been also shown that not all phosphorylation of Tau contributes to aggregation but rather the spatial domain of phosphorylation sites on Tau have an impact on the aggregation. For instance, the phosphorylation at Ser241 and Ser262 on the repeat domain of Tau prevents the aggregation [53], and in Sf9 cell culture, Tau is not aggregated despite the high concentration and extensive hyperphosphorylation of the protein, presumably due to the additional contribution of other factors that promote Tau aggregation [54]. Furthermore, regardless of the phosphorylation status, truncated Tau fragments containing the repeat domain are highly prone to aggregation. Human AD brains and rat transgenic models burdened with Tau fragment 151–391 develop Tau aggregation and neurofibrillary pathology [55]. Another Tau fragment, 255–368, formed by cleavage of an asparagine endopeptidase or long fragments (e.g., Tau 1–368 or 1–421), also causes neurofibrillary pathology in vitro [56,57]. In a cell model of tauopathy that expresses the ΔK280 (a repeat-domain-mutant), Tau accelerates robust aggregation and neurodegeneration [58,59].

Of note, pre-formed PHFs can act as external seeds to accelerate the aggregation in cultured cells without an endogenous nucleation step [42]. Similarly, when preformed PHFs are injected into the mouse brain, Tau seeds initiate widespread tauopathy and cause neuronal loss in a time-dependent manner [60,61].

### 2.4. Toxicity of Tau

**Figure 2:**
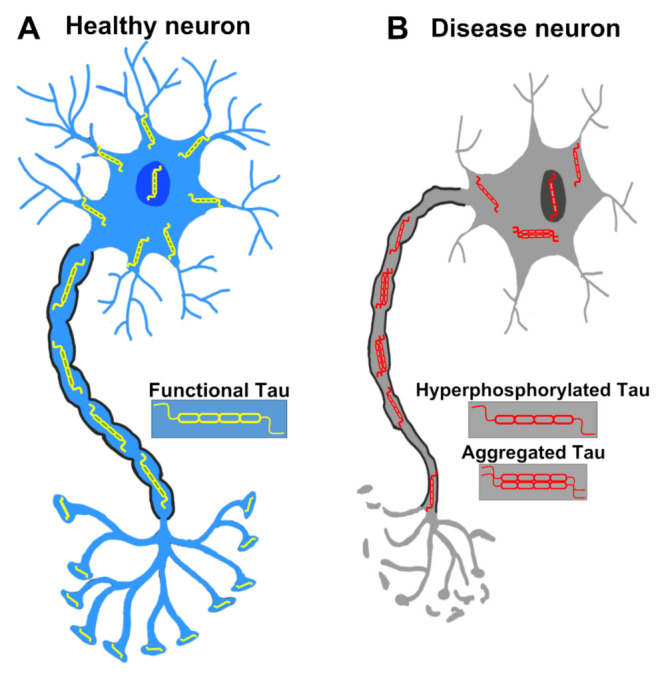
A healthy neuron and a pathological neuron in tauopathy. (**A**) In a healthy neuron, Tau is usually found in axons but also located in dendrites, the nucleus, the plasma membrane and synapses. (**B**) In the tauopathy condition, Tau undergoes PTMs such as hyperphosphorylation, resulting in the detachment from microtubules and the formation of aggregates. Tau dysfunction causes many neurodegenerative phenotypes such as pre- and post-synaptic decays, axonal degeneration, and eventually neuronal death.

Since there is a strong correlation between the presence of NFTs in the AD brain and the harshness of the memory impairment, NFTs have been suspected to be the direct culprit for neurodegeneration. However, the formation of NFTs turned out to be neither necessary nor sufficient to provoke neurodegeneration. For example, neurons with NFTs may live more than a decade in AD patients [62], which is in line with the observations from transgenic mouse models [63]. Neurons died before the formation of NFTs, and NFT-bearing neurons can survive without cellular activity defect [63]. Remarkably, in two reversible Tau expression transgenic mouse models, rTg4510 and ΔK280, which express human Tau^P301L^ mutation and pro-aggregation mutant, respectively, the decline in cognitive function was halted or even reversed after switching off Tau expression, regardless of the presence of NFTs. This finding strongly suggests that NFTs are not sufficient for the neurodegeneration [64,65,66]. Therefore, the toxicity that leads to neurodegeneration of Tau species is still under debate. Nonetheless, recent studies have indicated that Tau oligomers are the *bona fide* toxic species for neurodegeneration in tauopathy. Levels of soluble Tau oligomers in AD and PSP brains are significantly elevated [67,68], and a host of in vitro data has shown that Tau oligomers are neurotoxic [69,70,71]. It is also feasible that instead of being directly harmful, NFTs may alleviate the toxicity of Tau oligomers and thus protect neurons from degeneration [72]. However, it is still plausible that NFTs may be toxic by sequestering and occupying the cytosol space and thus inducing other cellular defects in the long term [73]. The normal neuron and pathological neuron in tauopathy are briefly depicted in Figure 2.

### 2.5. Clearance of Tau

Clearance of excessive and pathological Tau is a critical step for neurons to maintain the homeostasis of Tau protein and prevent Tau toxicity. Tau protein is cleared mainly via proteasome- and autophagy-dependent pathways. The general structures and function of the proteasome and autophagy are extensively reviewed elsewhere [74], and only Tau-related issues will be briefly covered in this review.

#### 2.5.1. Tau Is Degraded by the Proteasome

Tau is subject to degradation by the proteasome in an ubiquitylation-dependent or -independent manner. Tau can be ubiquitylated in vitro [75,76], and the modification at K6 inhibits ubiquitin-dependent degradation by the 26S proteasome contributes to the formation of NFTs [77]. Moreover, the impaired function of the 26S proteasome has been reported in the rTg4510 mouse model where accumulated Tau^P301L^ binds to the 26S proteasome and inhibits its hydrolyzing activity against ubiquitinated proteins [78]. Nonetheless, through different approaches, such as ATP depletion, ubiquitylation-deficient cells, knockdown of a 19S proteasomal regulator subunit, and in-vitro ubiquitylation studies, it was shown in several cell models that ubiquitylation was not required for Tau degradation by the proteasome 20S [79].

#### 2.5.2. Tau Is Degraded by Autophagy

Autophagy is a process known as “self-eating” that degrades substrates by the lysosome. There are three forms of autophagy: macroautophagy, chaperone-mediated autophagy, and microautophagy. The most common and well understood is macroautophagy [80] (hereafter, “autophagy” in short). An electron microscopic analysis of brain tissues from confirmed AD patients revealed that autophagic vacuole accumulates in dystrophic neurites and is correlated with the presence of NFTs [81]. In line with this clinical observation, a large number of studies have provided evidence that Tau is significantly degraded by autophagy. Cathepsin D is a protease that degrades Tau in the lysosome. When cathepsin D was added to the rat brain homogenates, Tau degradation was observed [82], and depletion of cathepsin D advanced Tau-induced neurotoxicity in a *Drosophila* tauopathy model [83]. In addition, when hippocampal slices were treated with chloroquine, a chemical well-known to raise lysosomal pH and inhibit autophagic flux, Tau protein levels (especially the PHF1 form) were significantly increased [84]. When the autophagy gene *Atg7* was deleted in forebrain neurons in a mouse knockout model, accumulation of phosphorylated Tau, together with NFT formation, was observed with age-dependent neurodegeneration [85]. All of these studies collectively indicate that autophagy plays a key role in mediating Tau clearance.

Mutations of Tau that are covered in this review are summarized in Figure 1B.

## 3. *Drosophila* Tauopathy Model

### 3.1. Advantages

*Drosophila melanogaster* has been extensively used as an excellent model system due to their short lifespan, small size, and increased propagation. In addition, *Drosophila melanogaster* allows in-depth reverse genetic studies and large-scale forward genetic screening [86]. Approximately 70% of the genes related to disease conditions in mammals are also present in *Drosophila* [87]. Generation of loss-of-function mutants or transgenic lines for the desired gene of interest is relatively easier than other model systems [88]. Based on these advantages, *Drosophila* has been an attractive model system for studying human neurodegenerative disorders, including tauopathies [89,90,91,92], helping us to understand the molecular mechanisms underlying neurodegeneration phenotypes.

### 3.2. Current Drosophila Tauopathy Models

*Drosophila* has a single homologous gene to human Tau in the genome. Its protein contains five microtubule-binding domains, which are 46% and 66% similar to 3R and 4R of human Tau, respectively [93]. As a well-established genetic model, nearly 100 *Drosophila* Tau models, using at least 37 human Tau constructs, have been generated [90] (Table 1). Different human Tau isoforms, as well as numerous Tau mutants and truncated variants, were overexpressed under pan-neuronal- or eye-specific promoters using a GAL4/UAS system, a well-established binary gene expression system in *Drosophila* [94]. Although *Drosophila* tauopathy models mostly do not show tau aggregation, Tau is found phosphorylated [14]. In addition, numerous tauopathy-related phenotypes can be observed in these flies, such as rough eye phenotypes (REP), neurodegeneration (ND) in the brain and eyes, learning and memory defects (LMD), reduced lifespan (RL), neuromuscular junction (NMJ), and locomotion defects. These phenotypes can be scored simply and quickly and have been successfully used in large-scale screens for searching modifiers of Tau pathology [92]. Thus, these fly models provide a versatile genetic tool to explore the vast diversity of tauopathies. The representative *Drosophila* tauopathy models and their phenotypes are listed and summarized in Table 1. (More information about *Drosophila* models can be found at https://flybase.org (accessed on 4 August 2021)).

### 3.3. Representative Assays

#### 3.3.1. REP

REP is the most commonly used phenotype for screening of molecular players in *Drosophila* because it provides a visible phenotype that can be easily quantifiable [110], hence making *Drosophila* useful for enhancer or suppressor screens of Tau toxicity [96,110,111]. Expression of the Tau gene in the eyes using eye-specific GMR-GAL4 generates roughening of eyes with reduced eye size due to retinal degeneration as a readout for Tau toxicity [95,112]. *Drosophila* REP screening was applied to identify orthologs of candidate risk genes using data from a genome-wide association study-AD (GWAS-AD) data involved in Tau neurotoxicity. The screening identified *Amph*, *p130CAS*, *Eph*, *Fak,* and *Rab3-GEF* as modifier genes [111]. Apart from genetic screening analysis, REP was also useful in analyzing the extent of neurotoxicity of different phosphorylation forms of Tau protein [113] and assessing electrophysiological properties of Tau using electroretinogram recordings [114].

#### 3.3.2. Neuronal Cell Death and Neurodegeneration

A shrunken brain, which is the consequence of cell loss in the brain, is the common feature of many neurodegenerative diseases, including tauopathy [115]. Therefore, neuronal cell death is examined regularly as one of the most representative phenotypes when a tauopathy animal model is generated. Tauopathy flies show increased vacuolar phenotype either in the brain (*Elav-GAL4*) or in the eye (*GMR-GAL4*) for neuronal degeneration due to neuronal cell loss. This phenotype can be visualized by immunohistochemical and histological methods using hematoxylin staining for vacuoles or TUNEL (Terminal deoxynucleotidyl transferase dUTP nick end labeling) assays for apoptotic cells in the tauopathy models [89,97,99,101,103].

#### 3.3.3. Learning and Memory Assays

The *Drosophila* mushroom body (MB) is the functionally homologous brain structure to the hippocampus of mammals, which is the epicenter of regulating learning and memory [116,117,118,119,120]. Expression of human Tau using a pan-neuronal driver (*Elav-GAL4*) affects olfactory learning and memory functions in *Drosophila* [105]. Restricted Tau expression to MBs also results in defects in learning and memory that were determined by testing responses to attractive and repulsive odors in Tau-overexpressing flies [121]. Similarly, an aversive phototaxis suppression assay was used in *Drosophila* tauopathy models to measure learning and memory function [122].

#### 3.3.4. Lifespan

The ultimate consequence of Tau toxicity is the reduction of lifespan in tauopathy flies, which can be analyzed by counting the number of surviving flies. Under normal conditions, wildtype flies live approximately 60 to 90 days, and neuronal expression of Tau isoforms and mutants reduce lifespan according to their toxicity [89,123]. Apart from the lifespan assay, pan-neuronal expression of Tau also leads to pupal lethality, which can be analyzed for Tau toxicity [102]. Overall, these phenotypes can be used to identify genetic modifiers of Tau toxicity.

#### 3.3.5. NMJ and Locomotion

The larval NMJ is a widely analyzed structure used to understand the mechanisms of synapse formation, growth, and maintenance [124,125]. The most commonly used approach for NMJ analysis is to drive expression of the gene of interest (e.g., Tau) in larval motor neurons, using drivers such as *D42-GAL4* and *OK6-GAL4*, and then visualize its activity within readily accessible larval motor neuron axons and NMJ using assays for axonal transport and synaptic function, respectively [91]. Expression of 0N3R Tau in NMJ using *D42-GAL4* affects morphological and functional disruption of NMJ with reduction in size and numbers of boutons, as well as reduction in mitochondria at the synapse [126]. In addition, hyper-phosphorylated Tau strongly affected vesicle motion in axons and synaptic function [102]. Synaptic abnormalities led to defects in larval locomotion, and *Drosophila* AD larvae expressing Aβ42, or Tau exhibited reduction of NMJ bouton number and larval locomotion [92,127,128]. Hence, the locomotion behavior in conjunction with the NMJ phenotype is quite useful in addressing neuronal morphological defects followed by associated behavioral changes upon expression of diverse Tau species and manipulations of Tau modifiers.

#### 3.3.6. Transposon Mobility

Transposable elements (TEs) are known as ‘jumping genes’ and constitutes approximately 45% of the human genome. Transposable elements are the mobile genetic sequences seen in all the eukaryotic genomes [129]. Somatic transposition of TEs, retrotransposons, which mobilize through an RNA intermediate—are observed in human brains [130,131,132], mouse [133], and fly models [134]. In addition, TEs have been also shown to carry transcriptional control elements that regulate the transcription of their own genes as well as neighboring host genes, which was intensively reviewed in a recent review [135]. Recent studies of TEs in *Drosophila* have shown TE activation to be in association with Tau pathology in AD [136]. They also showed that Tau alters TE activity in the *Drosophila* brain expressing human wild-type or mutant Tau, suggesting that genomic instability in Tau-mediated AD mechanisms occurs due to TE activation. Similarly, transposable element dysregulation was identified as a key mediator of neuronal death in tauopathies, where heterochromatin decondensation and reduction of piwi and piwi-interacting RNAs (piRNAs) drive transposable element dysregulation in tauopathy [137].

### 3.4. Limitations

Despite many advantages of *Drosophila* as a tauopathy model, a few limitations need to be considered. First, compared to other vertebrate species, such as mice, the anatomy of the *Drosophila* brain is quite different from that of the human, in that brain connectivity is relatively simple and asymmetric [138,139]. Second, established behavioral assays are relatively simple and limited to directly addressing in-depth cognitive function [140]. Lastly, the lack of some critical organs/tissues homologous to those of vertebrates that may contribute to tauopathy, such as the adaptive immune system, closed blood circulatory system, and blood-brain barrier, might cause inconsistent and unpredictable results when applied to humans [141,142].

### 3.5. The Utility of the Drosophila Tauopathy Models and Their Translational Applications

A study from the Drosophila tauopathy model showed that the PAR-1 kinase can trigger tau toxicity by directly phosphorylating Tau at Ser262 and Ser356 [99]. This phosphorylation is an important step for the activity of downstream kinases, such as GSK-3β/sgg and Cdk5, to phosphorylate other sites in Tau and generate phosphoepitopes in diseased conditions [99]. Such phosphorylation of Tau protein may cause dysfunction of microtubule stabilization and neurodegeneration, demonstrating that phosphorylation of Tau can occur in a sequential manner dictated by previous phosphorylation events [97]. Pharmacological inhibition of GSK-3β by treating with SB415286 and lithium [143,144,145] promotes synapse formation and lifespan, respectively. Using Elav-GAL4/UAS-WT or hTau^R406W^ Drosophila tauopathy models, nicotinamide mononucleotide (NAD) adenylyl transferase (NMNAT) were shown to be involved in a neuroprotective role of rescuing Tau^R406W^ mutant phenotypes by clearing toxic phosphorylated Tau proteins and facilitating proper neuronal function [122]. Apart from GSK3β signaling regulated by Insulin signaling pathway, other pathways JAK/STAT signaling pathway [146], and Nuak1, an AMP-activated protein kinase (AMPK)-related kinase [147] were identified in causing hyperphosphorylation and Tau toxicity. In addition, genes involved in regulators of actin network, viz, cheerio (a fly ortholog of filamin), chd64 (a fly ortholog of transgelin-3), jaguar (a fly ortholog of myosinVI), paxillin, were identified as modifiers of the Tau^V337M^ [148].

Ambegaokar and Jackson (2011) carried out one of the most exhaustive genetic screens using P-element insertion collections and identified a loss-of function collection consisting of 920 genomically mapped lethal P-elements [149]. In total, 1905 lines were screened and 37 modifiers of tau toxicity regulating autophagy, the cell cycle, and gene expression as well as phosphorylation were identified. Recent studies have also investigated the ability of small therapeutic compounds using a custom chemical library to improve tau-induced rough-eye phenotype in a Drosophila melanogaster model of frontotemporal dementia (FTD) and showed that Ro 31-8220, a potent inhibitor of PKCα, improved the rough-eye phenotype, reduced phosphorylated Tau species in vitro and in vivo, reversed Tau-induced memory impairment, and improved the fly motor functions [150].

As invaluable resources, the UAS-dsRNAi line libraries that cover more than 90% of the entire fly genome can be utilized for Tau regulator screening (Vienna Drosophila Research Centre, The Harvard Drosophila RNAi Resource Project, NIG-FLY Stock Centre and Bloomington Stock Centre), where various UAS-dsRNAi flies can be screened to identify the candidates involved in regulating Tau toxicity by crossing with UAS-Tau-expressing transgenic flies. Tissue specific knock down of UAS-dsRNAi in flies expressing UAS-Tau will give a clear picture in mediating Tau pathogenesis and its interaction with Tau proteins. For example, RNAi functional screening in Drosophila models of human Tau-mediated neurodegeneration found that the downregulation of the Drosophila REEP1 homolog enhanced Tau toxicity with increased formation of Tau insoluble aggregates [151]. They further showed that the overexpression of either the Drosophila or the human REEP1 protein was able to revert these phenotypes and promote neuronal resistance to ER stress. Another genetic screening using GMR-GAL4/UAS-hTau^V377M^ transgene have identified 16 enhancers and 8 suppressors of Tau toxicity which encode kinases and phosphatases as the major determinants of neurotoxicity in vivo [110]. In addition, a genetic screen for modifiers of 2N4R hTau toxicity has identified components of the ERK/MAPK and p38/MAPK pathways [149]. More specifically, a genetic modifier RNAi screening designed to validate AD susceptibility genes have identified cindr, fit1/2, and aret, the fly orthologs of the human CD2AP, FERMT2, and CELF1, respectively, as Tau modifiers implicated mainly in cell adhesion, together with identification of ITGAM and ITGA9, a fly homolog of the human integrin adhesion receptors [152]. This genome-wide genetic screening strategy can be modified by beginning the screening with a Drosophila genome-wide UAS-miRNA library followed by another RNAi screening for a selected set of identified miRNA-targets, yielding an E3/E4 ubiquitin ligase UBE4B as a novel tauopathy modifier to clear accumulated wild type Tau protein through autophagy [92].

## 4. Zebrafish Tauopathy Model

### 4.1. Advantages

The zebrafish is a small vertebrate animal model with high generation capacity [153]. Similar to other vertebrates, the anatomical structure of the zebrafish central nervous system is conserved, divided into the forebrain, midbrain, hindbrain, and the spinal cord, together with optical transparency at the larvae stage for superb observation capability [154,155]. Zebrafish and humans share approximately 70% similarity at the genome level, with 84% of counterpart genes related to human genetic diseases, including neurodegenerative diseases found in the zebrafish genome [156]. In addition, transgenic zebrafish can be efficiently generated with the Tol2 transposase system using specific tissue promoters by conventional expression [157] or conditionally using GAL4-UAS system [158] or Cre/loxP system [159]. Furthermore, targeted gene knockouts, such as Zinc Finger Nucleases (ZFNs), Transcription Activator-like Effector Nucleases (TALEN), and recently Clustered Regularly Interspaced Short Palindromic Repeats (CRISPR)-Cas9 [160], aided by a number of fluorescence-based transgenic lines, allow for the creation of disease models with high productivity [161].

Drug screening for neurodegenerative diseases including tauopathy requires a high-throughput in-vivo screening platform with large-scale screening capability because of the intricate etiologies of neurodegenerative diseases [162]. Zebrafish can provide a unique opportunity for the phenotype-based large-scale high-throughput screening in larval, as well as adult stages as a vertebrate animal model [163,164,165]. Indeed, through zebrafish chemical screening, several drugs have been discovered and translated into clinical trial [166]. For example, Lorcaserin, an FDA-approved serotonin agonist, was identified from a drug screening using *scn1* mutants to reduce the seizure severity and/or frequency in five Dravet syndrome patients [167].

### 4.2. Current Zebrafish Tauopathy Models

In zebrafish, one *MAPT* in humans is duplicated as paralogous genes *mapta* and *maptb*: *mapta* can be spliced into isoforms with four and six tubulin-binding repeats (4R, 6R), while *maptb* is mostly spliced into 3R isoforms [168]. Both paralogous genes are predominantly expressed in the CNS, with *maptb* largely expressed in the trigeminal ganglion and dorsal sensory neurons during early embryogenesis [168].

Since 2002, several zebrafish genetic tauopathy models have been reported using different strategies (Table 2). Among them, the most acknowledged model is the one from Haass group, in which the human Tau^P301L^ mutant form was overexpressed under pan-neuron-specific *HuC* promoter by utilizing a Gal4/UAS-based bidirectional expression system [169]. This transgenic zebrafish model exhibited pathological tauopathy characteristics like multiple pathological Tau-specific phosphorylation, defective motor axons/synapses, neuronal cell death in the spinal cord, the escape response defect at larval stages, and NFT formation at young adult stages [169]. Another zebrafish tauopathy model from the Rubinsztein group successfully confirmed the pathogenicity of Tau A152T variant (Tau^A125T^) as a risk factor for FTD and AD by generating a novel transgenic line overexpressing this human Tau variant using the Gal4/UAS system [170]. This Tau^A125T^ transgenic model also exhibits tauopathy phenotypes, including pathological Tau phosphorylation of AT270, PHF, and AT8, the presence of NFT, increased neuronal cell death, abnormal axonal pathfinding and branching, and the motility defect against touch response. In particular, the proteasomal activity was shown to be compromised in this model delaying Tau clearance, and induction of autophagy activity was suggested as a potential therapy [170]. Other zebrafish tauopathy models in which human Tau was directly expressed under a pan-neuronal promoter also showed frank phosphorylation of Tau, but other classical tauopathy phenotypes were either vague or undetectable in these models [171,172,173,174,175]. The reason for this inconsistency is not clear, but the expression level of Tau protein may be a key difference. Nonetheless, due to encouraging results of zebrafish as a tauopathy model, more transgenic lines are expected to follow, especially to take full advantage of zebrafish as a genetic tauopathy model. The current zebrafish tauopathy models and their phenotypes are listed and summarized in Table 2 (More information about zebrafish can be found at https://zfin.org (accessed on 4 August 2021)).

### 4.3. Representative Assays

#### 4.3.1. Neuronal Cell Death

In the zebrafish, assays for apoptosis detection were well developed and described either in whole-mount or in section by TUNEL staining or by acridine orange staining that detects dead cells in live zebrafish embryos without fixation [174,176,177]. Several antibodies for detecting apoptotic pathways can also be applied to zebrafish tauopathy models, such as anti-cleaved caspase-3 antibody [176].

#### 4.3.2. Axonopathies

Axonopathies, such as dystrophic axons, axonal swelling, and demyelination, are some of the distinctive neuropathological clinical features of tauopathy [178,179]. In developing zebrafish, primary motor axons with a large diameter appear at ~11 h postfertilization from the spinal segments and protrudes ventrally [180]. During developmental stages, these axons can be visualized live with high resolution using motor neuron-specific transgenic lines such as *Tg(mnx1:GFP)^ml2^* and *Tg(insm1a: EGFP)^ntu804^* [181] or using motor axon-specific antibody, Znp1 [182]. The continuous development of motor neurons has been examined without missing crucial events in live embryo with time lapse imaging [183] and can be applied to examine Tau- or Tau modifier-induced axonal defects with great detail in a zebrafish tauopathy model.

#### 4.3.3. Locomotion

Neuron-specific expression of Tau in tauopathy models affects the nervous system, resulting in morphological and functional defects at multiple levels, and the locomotion phenotype is an essential assay that allows evaluation of those defects collectively. Zebrafish locomotion has been intensively utilized due to its small size and high-throughput capacity at both larvae and adult stages [184,185]. For convenience of researchers, various kinds of the commercially available apparatus and software, plus detailed protocols have been well developed specifically for analyzing the locomotion in zebrafish, making such an assay feasible in zebrafish laboratories on a regular basis.

#### 4.3.4. Ubiquitin-Proteasome System (UPS) and Autophagy-Lysosomal Pathway (ALP)

In general, monomeric Tau protein is degraded by both 20S and 26S UPS [186,187,188,189], but such UPS is likely unable to clear aggregated Tau but is instead impaired by Tau toxicity [78,190]. In the zebrafish tauopathy model, apart from conventional methods such as western blotting for UPS markers, the UPS activity can be easily monitored in vivo by injecting Ub-R-YFP (substrate tagged with YFP, known to be degraded by the UPS) construct into embryos [191]. Thus, the examination of UPS malfunction is worth considering when characterizing zebrafish tauopathy phenotypes. Similarly, the ALP, another degradative pathway for Tau protein, can be visualized in vivo by expressing GFP-Lc3-RFP-Lc3∆G construct, as well as western blots of autophagy markers and specific inhibitor treatment in zebrafish models [192].

### 4.4. Limitations

Despite several advantages, the number of zebrafish tauopathy models is still limited compared to those of rodents, *Drosophila,* or *C. elegans* tauopathy models. More zebrafish tauopathy models can be developed and further characterized with a battery of tauopathy phenotypes in great detail in vivo. In particular, a few neurobehavioral assays in zebrafish during both larva and adult stages that may be directly relevant to tauopathy have yet to be applied to current and future zebrafish tauopathy models. For example, the anxiety level and a memory-based adaptive behavior, possibly disrupted in tauopathy, can be represented by simple neurobehavioral tests such as thigmotaxis/open field test and habituation of both larva and adult zebrafish, which can be devised for high-throughput screening.

### 4.5. The Utility of the Zebrafish Tauopathy Models and Their Translational Applications

Although zebrafish tauopathy models are limited, several new findings on pathological features and signaling factors for tauopathy have been discovered, and novel GSK3β inhibitors and Tau assembly modulators were validated as potential therapeutic targets for tauopathy using these zebrafish Tau models.

By combining human *Tau^P301L^*-overexrpessing zebrafish model with the “mitofish”, a transgenic zebrafish that was developed to study the dynamic and life cycle of mitochondria in real time in vivo, a profound mitochondrial transport deficit was visualized under a tauopathy condition [193]. This phenotype was rescued by overexpressing MARK2, the kinase that regulates the binding affinity of Tau to microtubules [194], thus directly demonstrating the impact of MARK2 on axonal transport in a Tau-dependent manner in vivo [193]. Similarly, the behavior of microglia in a tauopathy disease context in vivo was investigated by combining human *Tau^P301L^*-overexpressing zebrafish model with the *Tg(apoe:EGFP)* that marks the microglia [195]. Compared to the non-disease condition, the microglia in the zebrafish tauopathy model became highly mobile and dynamically changed their morphology with a fewer and shorter branching: more interestingly, the phagocytosis process by the microglia engulfing apoptotic neurons was also observed in real time, and furthermore, the genetic ablation of microglia increased the Tau hyperphosphorylation level, suggesting that microglia possesses a neuroprotective role in the tauopathy condition [195]. Also, as traumatic brain injury (TBI) is a conspicuous risk factor for dementias including Tauopathies, subjecting a novel TBI paradigm to the human *Tau^WT^* zebrafish larval model increased neuronal death and Tau inclusion of which phenotypes were rescued by dynamin inhibitors or anticonvulsant drugs [175], suggesting that the seizure activity has the strong impact on prion-like seeding and spreading of Tau following TBI.

Potential candidate factors that may modulate tauopathy were functionally validated using zebrafish tauopathy models. For example, dysregulated signaling of brain-derived neurotrophic factor (BDNF) is strongly associated with neurodegenerative diseases [196], but how BDNF signaling affects tauopathy or vice versa remains unclear. By exploiting the human *Tau^P301L^* zebrafish model, it was shown that the BDNF level was reduced and associated with axonal developmental defects, but not neuronal death, in the tauopathy condition, since exogenous BDNF supplementation was able to rescue the primary axonal growth but had no effect on Tau-induced apoptotic cells [197]. In addition, HS3ST2 (heparan sulphate glucosamine 3-O-sulphotransferase 2), also known as 3OST2, and REG-1α (regenerating islet-derived 1α) are two new factors that were shown to be critical for the abnormal phosphorylation of Tau in the *hTau^P301L^* zebrafish model [198]. A loss of function of *HS3ST2* result in an apparent inhibition of abnormal Tau phosphorylation in the brain and the spinal cord, leading to a complete rescue of axonal length and touch response phenotypes [198]. Reversely, overexpression of REG-1α increased tau hyperphosphorylation through the AKT/GSK3β pathway in the same zebrafish tauopathy model [199]. In terms of testing a Tau aggregation modulator, FKBP52 (FK506-binding protein with a molecular mass of ~52 kDa) was validated as a new player that is potentially applicable for therapeutics [49]. This immunophilin protein directly interacted with Tau protein and induced the formation of Tau oligomers in vitro, with EM analysis showing that FKBP52-induced oligomers assembled into filaments. In the human *Tau^P301L^* zebrafish model, knockdown of *FKBP52* debilitated the pathological Tau activity and recovered axonal outgrowth and branching in defective motoneurons, confirming the functional implication of FKBP52 in modulating Tau conformational change and assembly in vivo [49].

To take advantage of zebrafish as an in vivo animal model for chemical genetics, zebrafish tauopathy models were also utilized for candidate small molecule validation. GSK3β, one of the main kinases that phosphorylates Tau, is therefore considered as a key therapeutic target for tauopathy. The human *Tau^P301L^* transgenic zebrafish was adopted to validate the efficacy of a newly developed GSK3β inhibitor, AR-534, confirming its stronger effect on diminishing pathologic Tau phosphorylation with a high selectivity and good bioavailability in vivo than SB-216763 and SB415286, two well-known GSK3β inhibitors [169,200]. In addition, a few FDA-approved clinical drugs were tested in the human *Tau^P301L^* zebrafish model. Given that HS3ST2 induced abnormal Tau phosphorylation as aforementioned, surfen and oxalyl surfen are small molecules harboring heparan sulfate antagonist properties and well tolerated in clinical setting in cancer treatment [201,202]. These two small molecules were capable of mitigating Tau hyperphosphorylation and rescuing spinal motoneuron defects, leading to recover the touch escape response in the zebrafish *Tau^P301L^* tauopathy model [203]. Sildenafil or tadalafil, an inhibitor of phosphodiesterase 5, widely used for erectile dysfunction and pulmonary hypertension treatment [130], was also shown to be a promising strategy to prevent Tau toxicity: by activating cGMP (cyclic guanosine monophosphate)-dependent protein kinase, the stimulated proteasome activity reduced the level of Tau protein and decreased the associated morphological abnormalities in the zebrafish human *Tau^A152T^* model [204].

Finally, zebrafish tauopathy models can be also applied for mid- to high-throughput in vivo chemical library screening. An embryonic zebrafish tauopathy model that transiently overexpresses Tau-GFP fusion was used to screen 400 herbal extracts using neuronal death as an in vivo readout [205]. 45 out of 400 extracts were confirmed to be effective in reducing Tau-induced neuronal death, and among them, the extract from *Tripterygium wilfordii* stem showed the highest effect. HPLC analysis showed that its major compound was epicatechin that activated Nrf2-dependent antioxidant responses and thus abated Tau-induced neuronal death [205]. Of note, overexpression of multiple signaling factors such as Bcl2-L1 or GDNF was also capable of effectively protecting neurons from Tau-induced apoptosis in the same zebrafish tauopathy model [173,205].

## 5. *C. elegans* Tauopathy Models

### 5.1. Advantages

*Caenorhabditis elegans* (*C. elegans*) is a small invertebrate genetic animal model that possesses a high reproduction ability (once every 3–4 days) and a short lifespan (approximately 2–3 weeks) [206,207]. A major advantage of *C. elegans* is that desired phenotypes can be assessed faster than any other model organisms [208,209]. Regardless of its simplicity, *C. elegans* is a multicellular organism composed of the brain, pharynx, intestine, gonads, muscle, and rectum. Furthermore, the transparency of its body during its lifetime facilitates live imaging with the aid of fluorescent protein reporters under tissue-specific promoters, including neuronal promoters. *C. elegans* has 302 lineage-identifiable neurons, thus increasing the accuracy of neuronal analyses while reducing the complexity [210]. In the context of neurodegenerative disorders research, neuronal cell death or protein aggregation can be easily observed and quantified, allowing single cell-lineage analysis for a phenotype at an organism level [211]. Practically, the maintenance of *C. elegans* is very convenient due to its small size even in the adult stage (~1 mm) and the ability to be stored in liquid nitrogen and recovered years later [212].

*C. elegans* have 60–80% orthologous genes with humans, with more than half of them associated with human diseases [213]. Along with the completed sequenced genome [214], the compendium of neuronal connectomes and the whole neuronal expression map by the CeNGEN project of *C. elegans* using state-of-the-art technologies are available. These tools help to understand neuronal function with single cell resolution at the organism level [210,215]. Transgenic *C. elegans* models for neurodegenerative diseases can be generated easily by traditional microinjecting transgene DNA [216] or biolistic bombardment using DNA-coated gold particles to integrate exogenous DNA with high efficiency [217]. Altogether, *C. elegans* is a still convenient and promising in vivo system for modeling human mutation-associated diseases while maintaining favorable features of in vitro systems.

### 5.2. Current C. elegans Tauopathy Models

*C. elegans* has two PTL-1 isoforms, PTL1a and PTL1b, homologous to human Tau. The two isoforms are differentiated by the number of tandem repeats (5 repeats in PTL-1a and 4 repeats in PTL-1b) and share a homologous microtubule-binding region with mammalian Tau [218]. To date, nearly 20 lines of *C. elegans* tauopathy models from seven groups have been established by taking advantage of the convenient genetic manipulation. Various isoforms (0N4R, 1N4R, 2N4R), as well as wild type (WT) and mutation forms (P301L, V337M, R406W, A152T, V363I, V363A, T231E, K274/281Q, ∆K280, pseudo-hyperphosphorylation form) of human Tau were expressed under pan-neuronal or touch-neuronal promoters in the transgenic worms (Table 3). In these tauopathy models, most of the pathological features of human tauopathy, such as Tau phosphorylation, axonal degeneration, neuronal loss, synapse dysfunction, and mitochondria defect, were in an age-dependent manner. Typical *C. elegans* behavioral phenotypes, including uncoordinated moving, touch response, or thrash rates, were affected to different degrees of severity depending on the expressed forms of human Tau species [59,219,220,221,222,223,224]. The representative *C. elegans* tauopathy models and their phenotypes are listed and summarized in Table 3 (More information about *C. elegans* can be found at https://wormbase.org (accessed on 4 August 2021)).

### 5.3. Representative Assays

#### 5.3.1. Lifespan

Due to the short life cycle of *C. elegans* (reproduction, every 3~4 days; lifespan, 2~3 weeks), different stimuli that influence survival are widely used [225]. For example, genetic manipulations or temperature effects on the lifespan of *C. elegans* can be tested on the solid bacteria agar plate, whereas the treatment of diet compounds or chemicals can be more feasible in liquid media [226,227].

#### 5.3.2. Neuronal Death

Similar to *Drosophila* or zebrafish tauopathy models, neuronal cell death is one of the indispensable pathological phenotypes in *C. elegans* tauopathy models. Various staining techniques, such as TUNEL, acridine orange, or immunostaining, can be used to visualize this phenotype in worm models [228].

#### 5.3.3. Axonal Defects

In *C. elegans* tauopathy models, the simple morphology of cells and tissues labeled with fluorescence markers have made it possible to use live imaging to examine pathophenotypes of motor and mechanosensory axons in great detail and to assess axonal discontinuities, morphological abnormality, synapse defects, and faulty commissure [229,230,231,232].

#### 5.3.4. Behavior Phenotypes

Generally, behavioral phenotype assays to test Tau toxicity in *C. elegans* are mobility-based [233]. In the thrashing assay, a well-established motility-based assay, the frequency and lateral swimming movements of worms placed in liquid media are measured [234]. Other mobility-dependent indexes, such as uncoordinated movement, distorted wave motion, or locomotion speed, are also commonly used as behavioral assays in *C. elegans* models to test effects of drugs, chemicals, or genetic mutations [235,236].

### 5.4. Limitations

Ironically, the aforementioned advantages of *C. elegans* can be potentially disadvantageous in tauopathy research under certain conditions. For instance, the small size and limited number of neurons of *C. elegans* can be a drawback in biochemical approaches because the small number of touch neurons overexpressing human Tau (a total of only six neurons) causes difficulty in detecting Tau protein abnormalities such as thioflavin staining [224]. In addition, although *C. elegans* carries 60–80% orthologous genes with human and mutant line generation in *C. elegans* is straightforward, only 30% of genes of *C. elegans* can be mutated because of high lethality [237]. Although gene-silencing techniques such as RNAi can be complemented to knock down the gene of interest, such knockdowns in the *C. elegans* nervous system are often refractory [238,239,240], thereby hampering the tauopathy modifier studies. Furthermore, lack of features of vertebrate neurons, such as DNA methylation or myelination of axons, in addition to absence of several defined vertebrate organs, such as brain and circulatory system, may lead to an inconsistency in both physiological and pathological outcomes of tauopathy models of *C. elegans* and human cases and in screening results for tauopathy drugs or compounds [241]. Finally, despite recent studies showing the ability of this model to learn and remember experiences [242,243], it may be too simple to deduce the cognitive behavioral phenotypes observed in mammalian neurodegenerative disease.

### 5.5. The Utility of the C. elegans Tauopathy Models and Their Translational Applications

Even though *C. elegans* is a very simple model and appears to be far from being an alternative for vertebrate models as a clinical testing system for neurodegenerative diseases, the *C. elegans* model research for tauopathy, covering the discovery of its characteristics and the identification of the drugs or therapeutic targets, has been quite informative and worth being validated in higher model systems before transferring to clinical trials.

For instance, to assess the significance of the Tau aggregation process in tauopathy, a pro-aggregation *C. elegans* model overexpressing human *Tau^ΔK280^* was examined in parallel with another transgenic line co-expressing anti-aggregation substitutions I277P and I308P of Tau that prevent β-sheet formation and subsequent aggregation (human *Tau^ΔK280^*, plus I277P and I308P) [59]. As a result, the pro-aggregation strain showed severe motility impairment, neuronal dysfunction as well as disturbed axonal transport of mitochondria which was not observed in the strain that co-expresses the anti-aggregation substitutions. Furthermore, several Tau aggregation inhibitors showed the ability to prevent Tau toxicity in the pro-aggregation strain, highlighting that inhibiting the Tau aggregate formation may be a potential target and Tau aggregation inhibitors could be encouraged to test in clinical trials [59]. Another example is the function validation of dihydrolipoamide dehydrogenase, one of the major metabolic enzymes of mitochondria [244]. In the human *Tau^W^*^T^ *C. elegans* model, suppression of this gene either by RNAi or inhibitor resulted in increased whole-body glucose levels and Tau phosphorylation, consistent with the observation in AD patients [245], suggesting that the disturbance in energy metabolism can significantly induce the neurotoxicity of pathological Tau [246]. Also, co-expression of wild type LRRK2, known as a genetic cause of Parkinson’s disease and some cases of Tauopathy [247], led to increased expression of several 60S ribosomal, mitochondrial, and V-type proton ATPase proteins without altering the redox status in the human *Tau^V337M^ C. elegans* model. Moreover, co-expression with mutant LRRK2 (G2019S or R1441C) showed similar effects additionally with increased protein oxidation and lipid peroxidation, revealing roles of the LRRK2 activity in a Tau-dependent context [248]. In addition, a selective loss of glutamatergic neurons was observed in a human *Tau^A152T^* overexpressing transgenic *C. elegans* [249]. In this strain, the glutamatergic nervous system was shown to be particularly vulnerable, largely due to necrotic cell death pathway. Genetic analysis also revealed this phenomenon occurred through several mechanisms, including type 9 adenylyl cyclase signaling, signaling through glutamate receptor complexes, aging-related signaling, and Ca^2+^ dyshomeostasis with increased Ca^2+^ released from the ER [249].

In a recent study, a “PTM-mimetics” *C. elegans* strain was generated to clarify the precise impact of PTMs on neural toxicity of Tau. This strain contained mutations of T231E that mimics phosphorylation of pathological epitope or K274Q and K281Q that mimic disease-associated lysine acetylation as a single-copy gene insertion by CRISPR-Cas9 genome editing [224]. This “PTM-mimetics” strain showed reduced touch response and exhibited neuronal morphological abnormalities in an age-dependent manner, as well as mitophagy defect in response to mitochondrial stress. By limiting Tau expression as a single-copy, not as overexpression, the decisive role of PTMs in the pathogenesis of tauopathy has been emphasized, thus providing a new perspective for the therapeutic approach [224].

Curcumin, one of the common used spices and ancient medicines, has been validated epidemiologically and currently tested for clinical trials of various diseases [250]. Interestingly, epidemiological studies showed that the prevalence of AD in India is less than in US, which may be explained by the curcumin-rich diet [251]. Based on this observation, Yasuo Ihara group have tested the effects of curcumin on *C. elegans* human *Tau^WT^* and *Tau^R406W^* models [252]. As a result, curcumin rescued not only the uncoordinated (Unc) phenotype but also the neuritic abnormalities of both human *Tau^WT^* and *Tau^R406W^* expressing worms. However, curcumin did not prevent Tau hyperphosphorylation nor the aggregation, but instead increased the level of acetylated α-tubulin, suggesting tubulin may play a critical role in controlling Tau toxicity [252]. The following study has disclosed the important of balanced expression of Tau and tubulin in tauopathy by examining several different strains of *C. elegans* expressing various levels of WT Tau and assessing the effect of the tubulin [253]. The knockdown of *tbce-1*, a human tubulin-folding cofactor E homologue, or α-tubulin led to enhanced tau toxicity in a Tau level-dependent manner. Thus, the neurotoxicity of Tau can be triggered by reducing tubulin levels, even in a very low Tau-expressing worm that showed no abnormal behaviors per se. More strikingly, co-incubation of purified tubulin was able to inhibit Tau aggregation in vitro. Therefore, the ratio of expression between Tau and tubulin may be a determinative character of the tauopathy cascade [253].

One of the obvious advantages of *C. elegans* tauopathy model is a large-scale genetic screening capability. Indeed, a RNAi library screening was conducted using human *Tau^V337M^ C. elegans* model: out of 16757 genes screened, 60 genes showed increased Unc phenotype in the Tau transgenic worm. Confirmed by loss-of-function studies, *aex-1*, *acr-14*, *lin-44*, *sir-2.3*, *pxn-1*, and *vap-1* were finally identified as enhancer factors of Tau toxicity, being novel candidate genes associated with tauopathy since human homologs were found [254]. Similarly, from a forward genetic screening using human *Tau^V337M^ C. elegans* model, recessive mutations of *sut-1* and *sut-2* partially suppressed the Unc phenotype, Tau aggregation and pathological features of tauopathy. SUT-1 protein was shown to interact with UNC-34 protein, while SUT-2 physically bound to ZYG-12, required for tau neurotoxicity. Moreover, mammalian SUT-2, which is MSUT2, was found markedly decreased in the post-mortem temporal lobe brain region of AD patients. In line with this, in vitro data also showed that high levels Tau led to increased expression of MSUT2 protein [255,256,257]. Taken together, screening of tauopathy *C. elegans* models allowed the identification of several conserved molecular pathways participating in Tau neurotoxicity, which are potentially utilized to be novel strategies for treating tauopathy.

## 6. Conclusions

**Figure 3:**
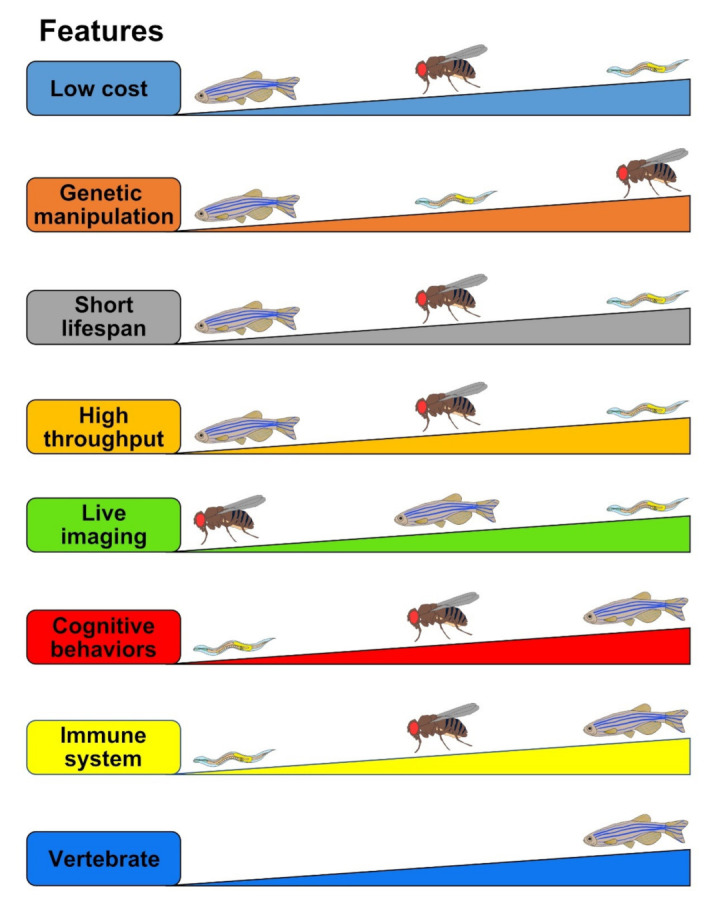
Comparisons of the strength and limitations of *Drosophila*, zebrafish, and *C. elegans* as tauopathy models. The evaluated criteria (“Features”) are represented as the thicker means the better. For example, in the Low cost feature, *C. elegans* stands in the first place which is the cheapest, followed by *Drosophila* in the second and zebrafish in the last.

The etiology of tauopathy is very complex and multi-faceted, and finding an efficient cure is imperative. Appropriate utilization of animal models that mimic essential pathophenotypes of tauopathy will reveal underlying mechanisms and identify key modifiers for disease prevention or novel drug target discovery. Despite their imperfections, the small genetic animal models discussed in this review (*Drosophila*, zebrafish, and *C. elegans*) have greatly helped scientists to appreciate diverse aspects of Tau biology as well as to understand detailed molecular pathways of Tauopathy (Figure 3, Table 4). Through their major advantages, including (1) low cost in infrastructure and maintenance, (2) short lifespan, (3) strong flexibilities of genetic manipulation, and (4) real-time, live imaging, and/or (5) high-throughput screen capability, tauopathy models of *Drosophila*, zebrafish, and *C. elegans* will continue to contribute to the discovery of a novel therapy for Tauopathy.

## Figures and Tables

**Table 1:** Drosophila tauopathy models. GMR-GAL4: eye-specific GAL4 driver; Elav-GAL4: pan-neural GAL4 driver; OK-GAL4: motor neuron-specific GAL4 driver; MB-GAL4: Mushroom Body-specific GAL4 driver; C161-GAL4: sensory neuron-specific GAL4 driver; D42-GAL4: motor neuron-specific GAL4 driver. hTauWT: human Tau wildtype; LMD: learning and memory defects; MT: microtubule; ND: neurodegeneration; NMJ: neuromuscular junction; pTL: pTau levels; REP: rough eye phenotype; RL: reduced lifespan.

Tau Isoform	Constructs (UAS)	GAL4 Driver	Phenotypes	References
*2N4R*	hTauWT	*GMR-GAL4, Ealv-GAL4*	REP, ND	[95]
	hTauP301L	*GMR-GAL4*	REP, ND	[96]
	hTauWT, hTauS2A hTauS11A	*GMR-GAL4*	REP, ND	[97]
	hTauWT:FLAG hTau^STA^:FLAG	*Elav-GAL4, OK107-GAL4*	ND, LMD	[98]
*0N4R*	hTauWT hTauV337M hTauR406W	*Elav-GAL4* *OK6-GAL4*	RL, ND TL, MT	[89]
	hTauR406W*/*S2A hTauR406W*/*S202A	*GMR-GAL4*	REP, ND	[99]
	hTauT111A*/*T153A hTauT175A*/*T181A hTauT199A*/*T217A hTauS202A*/*S205A hTauT212A hTauS214A hTauT231A*/*S235A hTauAP5 hTauS422A hTauS396A*/*S404A	*GMR-GAL4*	REP, pTL	[100]
	hTauS262A	*GMR-GAL4*	REP, ND	[101]
	TauAP	*Elav-GAL4, OK6GAL4*	MT defects	[102]
	hTauE14	*GMR-GAL4,*	REP, pTL, ND,	[103]
	hTauK44Q*/*R230Q hTau44–230	*GMR-GAL4*	REP, calpain activity	[104]
	hTau1–421	*Elav-GAL4*	RL, ND, pTL	[83]
	PH-Tau	*Elav-GAL4, MB-GAL4*	climbing, MB defects, LMD	[105]
*0N3R*	hTauWT	*C161-GAL4*	sensory neurons defects	[106]
	hTauWT	*D42-GAL4*	locomotion, axon, NMJ defects	[107]
	hTauWT	*Elav-GAL4*, *D42-GAL4*	pTL, MT stability defects	[108]
*dTau*	tau^EP3203^		MT stability defects	[109]
	tau^EP3597^		MT stability defects	[109]

**Table 2:** Zebrafish tauopathy models. hTau: human Tau; N/A: not applicable; TBI: traumatic brain injury; WT: wildtype; +: positive.

Tau Isoform (Mutation)	Promoter	Biochemical Phenotype	Biological and/or Behavioral Phenotype	Rescue	References
hTau 2N4R WT	*GATA-2*	- pS396/S404 (PHF)+ - NFTs	disrupted cytoskeletal filaments	N/A	[171]
hTau 0N4R WT	*eno2*	Tau accumulation	N/A	N/A	[172]
hTau 2N4R (P301L)	(UAS/GAL4) *HuC*	- pT231/S235, pT181, pS262/S356, pS396/S404, pS422, and pS202/T205+ - NFT formed	- neuronal cell death - shortened motor axons - defective locomotion	GSK3β inhibitor reduced Tau hyperphosphorylation	[169]
truncated hTau	*HuC*	AT8+	neuronal cell death	overexpression of Bcl2-L1, Nrf2, and GDNF rescued neuronal cell death	[173]
hTau 2N4R (P301L)	*HuC*	AT8+	N/A	N/A	[174]
hTau 2N4R (A152T)	(UAS/GAL4) *HuC*	- AT270-, PHF-, AT8+ - NFT formed	- defective motor axons (truncated, abnormal pathfinding branching) - neuronal cell death - delayed Tau clearance due to proteasome defect - defective escape response	activated autophagy rescued all phenotypes	[170]
hTau 2N4R WT	*eno2*	N/A	TBI induces - neuronal cell death - post–traumatic seizures - Tau inclusions	dynamin inhibitors/anti-convulsant drugs rescued TBI-induced Tau inclusions/cell death	[175]

**Table 3:** hTau: human Tau; N/A: not applicable; PHP: pseudohyperphosphorylated; Unc: uncoordinated; TAI: Tau aggregation inhibitors; ThS: thioflavin S; WT: wildtype; +: positive.

Tau Isoform (Mutation)	Promoter	Biochemical Phenotypes	Biological Phenotypes	Behavioral Phenotypes	Rescue	References
hTau 1N4R (WT, P301L, V337M)	*aex-3* (pan neuronal)	12E8, AT8, pT205, AT270, pT181, CP13, PHF-1, pS422+	- defective presynapse of cholinergic neurons - progressive axonal degeneration - neuronal cell death	- age-dependent Unc - reduced thrash rate	N/A	[219]
hTau 0N3R(WT), 0N4R(WT, P301L, R406W)	*mec-7* (touch neuron)	PHF-1, AT8+	- morphologic abnormalities in touch neurons - microtubule loss - non-apoptotic neuronal death	age-dependent touch response defect	HSP70 expression improved touch response	[220]
hTau 0N3R(WT, PHP)	F25B3.3 (pan-neuronal)	Tau5, AT180, PHF-1, AT8, TG3+	defective motor axons by PHP-Tau	progressive age-dependent Unc	N/A	[221]
- hTau 1N4R(V337M) + ∆K280 - hTau 1N4R(V337M) + ∆K280(I227P;I308P)	*aex-3* for 1N4R (V337M); *Rab-3* for ∆K280 (pan-neuronal)	- K9JA, 12E8, PHF1+, - ThS+	- axonal degeneration of GABAergic and cholinergic motor neurons - pharyngeal presynapse - synapse loss - defective mitochondrial transport	early-onset paralysis (1N4R(V337M) + ∆K280)	TAI reduced Tau expression/rescued the locomotion	[59]
hTau 2N4R (WT, A152T)	*Snb-1* (pan-neuronal)	- K9JA+ - MC1+	A152T showed: - acute neuronal disfunction - shorter lifespan - mislocalization of pre-synaptic proteins - distorted mitochondrial distribution and trafficking	early-onset paralysis (A152T)	TAI did not rescue paralysis	[222]
hTau 2N4R (V363I, V363A)	*aex-3* (pan-neuronal)	- SP70, AT180, AT8+	- defective neuromusculature - defective pharynx	- increased body bending - reduced lifespan	N/A	[223]
hTau 0N4R (T231E, K274/281Q)	*mec-7* (touch neuron)	N/A	- neuronal morphological abnormalities - suppressed paraquat-induced mitophagy	reduced touch sensation	N/A	[224]

**Table 4:** Summary of tauopathy models of *Drosophila*, zebrafish, and *C. elegans*.

	Strength	Limitation
**Mouse** (not covered in this review)	- mammalian model - phylogenetically close to human - almost 100% human homolog genes found	- costly in infrastructure and maintenance - time-consuming - large-scale studies are limited
***Drosophila***	- low-cost in infrastructure and maintenance - highly amenable to genetic manipulations - short lifespan - high-throughput screening feasible	- simple brain structure - relatively simple cognitive behaviors - lack of the adaptive immune system
**Zebrafish**	- a vertebrate animal model - highly homologous genes to human - live imaging - complete immune system - rapid development - high-throughput screening available	- limited cognitive behavioral assays - relatively expensive in infrastructure and maintenance (compared to *Drosophila* and *C. elegans*) - genetic tools yet to be comprehensive (compared to *Drosophila* and *C. elegans*)
***C. elegans***	- easy and low-cost in infrastructure and maintenance - highly amenable to genetic manipulations - live imaging - very short lifespan - high-throughput screening feasible	- less homologous genes to human - extremely simple cognitive behaviors - different anatomical systems (no brain structure and immune system, etc.)
